# Decoding Prejudice: Understanding Patterns of Adolescent Mental Health Stigma †

**DOI:** 10.3390/jcm14041394

**Published:** 2025-02-19

**Authors:** Sara Albuquerque, Ana Carvalho, Bárbara de Sousa, Leonor Pereira da Costa, Ana Beato

**Affiliations:** 1 HEI-Lab: Digital Human-Environment Interaction Labs, Lusófona University, 1749-024 Lisbon, Portugal; sara.albuquerque@ulusofona.pt (S.A.);; 2 School of Psychology and Life Sciences, Lusófona University, 1749-024 Lisbon, Portugal

**Keywords:** stigma, mental health, adolescents, cluster analysis

## Abstract

**Background/Objectives**: Mental health problems are a major cause of disability, impacting nearly 20% of adolescents. Nevertheless, they are hesitant to seek help because of stigma and fear of being labelled. Adolescents often have low mental health literacy and perceive mental health problems as personal failures. To address it, our study aimed to identify subgroups within the adolescent population based on mental health knowledge, social stigma, experiences of intergroup anxiety, and endorsement of stereotypes. **Methods**: This cross-sectional study included 182 adolescents (50.6% male) aged 10 to 17 years (M = 13.8, SD = 2.4). Participants completed an online survey comprising the Mental Health Knowledge Schedule, Attribution Questionnaire (AQ-8-C), Intergroup Anxiety Scale, and a scale regarding stereotypes towards people with mental health problems. Cluster analysis was used to identify the subgroups. **Results**: We identified three subgroups: (1) “Potential Advocates”, showing high mental health knowledge, low social stigma, low intergroup anxiety, and moderate endorsement of stereotypes; (2) “Ambivalents”, manifesting high mental health knowledge, moderate social stigma, heightened intergroup anxiety, and low endorsement of stereotypes; and (3) “Stigmatizers”, revealing low mental health knowledge, pronounced social stigma, moderate intergroup anxiety, and tendency to endorse stereotypes. **Conclusions**: The results highlight the multiplicity of perceptions regarding mental health and the pivotal role of knowledge, stigma, intergroup dynamics, and stereotypes in shaping attitudes. Implications for interventions targeting mental health stigma and fostering positive attitudes among adolescents are discussed, underscoring the importance of customised strategies to address the multiple needs and experiences characteristic of this developmental stage.

## 1. Introduction

Adolescence, the intermediary stage between childhood and adulthood, is characterised by profound physical, cognitive, emotional, and social transformations [1,2,3]. While early childhood development relies heavily on parental and community support, adolescence brings growing independence, as maturing individuals begin to take on more responsibility [4]. Nonetheless, this stage is frequently associated with a heightened susceptibility to mental health disorders [5], including depression, anxiety, eating disorders, and substance abuse, many of which emerge prior to the age of 24 [6].

Despite this increased risk of mental health disorders, adolescents face considerable reluctance to seek help that stems from multiple interconnected factors. These include stigma, fear of social judgment, decreased levels of mental health literacy, unawareness about available services, negative attitudes towards professionals, and concerns about confidentiality [7,8,9,10]. These challenges are exacerbated by limited awareness of mental health disorders and the expectation of adverse reactions from peers, family members, and educational personnel [11,12].

The stigma surrounding mental health can serve as a significant obstacle to seeking professional help [13,14,15,16], as it encompasses harmful stereotypical attitudes and behaviours that associate mental illness with personal weakness, unpredictability, or danger [17,18]. Such attitudes exist at all societal levels [19], from families and peers to media portrayals and even on those affected [20]. The intersection with gender, race/ethnicity, and socioeconomic status further influences stigma. Vulnerable groups, such as LGBTQ+ individuals or those from low-income backgrounds, often face compounded stigma, exacerbating obstacles to accessing support for mental health [21].

### 1.1. Adolescents and Stigma: Vulnerability and Consequences

Adolescents are especially susceptible to stigma, since social image, peer acceptance, and relationships strongly influence their self-esteem and identity [22,23,24]. Fear of judgment or ostracisation by their peers due to any form of deviation from perceived social norms and expectations (i.e., mental health problems), often leads them to hide struggles or avoid seeking help [25,26,27].

These fears are legitimate as negative mental illness attitudes are generally reinforced by adolescents [28]. Adolescents hold more stigmatised views of mental disorders when compared to the general population, contributing to barriers in academic, social, and personal spheres [29,30,31]. Mental health issues are often associated with personal weakness, failure, or unpredictability [28]. In a UK study of adolescents aged 14, the majority of descriptors for individuals with mental illness were derogatory, with terms like “nuts”, “scary”, and “isolated” dominating [32].

Stigma contributes to avoidance, social distancing, and discrimination [33]. Adolescents may engage in social distancing, where they may consciously or unconsciously withdraw from interactions with peers who exhibit signs of mental illness, fearing association with stigma or loss of social status [34]. They may likewise discriminate (using derogatory language or behaviours) towards their peers—in school, social gatherings, and online platforms—perpetuating harmful stereotypes, reinforcing the cycle of stigma further isolating individuals, and impeding their access to supportive networks [34,35]. Also, Kelly et al. [36] found that most high school students were hesitant to seek help from adults for peers in need, a reluctance influenced by societal attitudes and fear of discrimination associated with psychiatric labels. This aligns with Link and Phelan’s [37] concept of social stigma, which involves prejudiced attitudes and discriminatory behaviour towards individuals based on their social behaviour or awareness of their psychiatric labels and treatments. Furthermore, the stigma regarding mental health is particularly concerning in adolescence, since peer acceptance has a key role to play in the well-being of adolescents in general, and peers are often the first recourse for adolescents dealing with mental health problems because they prefer to seek informal help, i.e., from their friends [38].

Media representations, such as films portraying people with mental health problems as dangerous or abnormal, reinforce these perceptions [39]. Social media further complicates the issue, exposing adolescents to unrealistic standards of mental health and well-being, potentially intensifying feelings of inadequacy or shame [40]. Furthermore, while online platforms can provide support, where adolescents share information online with people, they feel that they can trust [41], they may over-rely on unverified online advice, heightening the risk of misinformation and inadequate coping strategies [42,43].

It is important to consider that social media is an important resource, often used by adolescents, to develop their identities and receive feedback from their peers [44]. Nonetheless, studies in Finland argue that excessive internet use causes exhaustion, depressive symptoms, and feelings of inadequacy, with depressive symptoms being more common in female adolescents [45]. It is relevant to consider that adolescents are attracted to social media because they can express themselves, get involved in a community, and find health information anonymously [46]. Facebook, the older Twitter, and Snapchat, among others, allow adolescents, especially those with mental health problems, to express themselves in a way that they might not be able to do in another environment [46]. Additionally, according to Carrier et al. [47], as adolescents express themselves, they begin to form empathetic communities, and this virtual empathy becomes related to feelings of social support.

Negative mental health attitudes can lead to marginalising people with these problems, discouraging help-seeking, and creating barriers to recovery, academic achievement, and social relationships [30,48,49]. Adolescents often attempt to manage mental health issues alone, further delaying intervention [50] and decreasing opportunities for early intervention [51]. Feelings of shame, self-blame, and fear of being perceived as weak compound these challenges, prolonging suffering and hindering recovery [30,52]. Additionally, endorsing stereotypes, including the belief that people with mental health problems are violent or incapable of decision-making, can lead to policies that prioritise restrictive practices, rather than prevention and community-based services [49,52]. Nevertheless, in a study by Robinson et al. [53], it was shown that stigma and trivialisation are highly prevalent on social media, and, considering that social interactions occur more frequently online, proactive campaigns must consider assessing and addressing both on social media.

### 1.2. Current Study and Objectives

Unaddressed mental health issues in adolescence can lead to long-term consequences for individuals and society (e.g., [54]) and mental-health-related stigma constitutes a primary barrier to seeking help and recovering (e.g., [49]). Also, adolescents hold more stigmatised views of mental health problems, when compared to the general population (e.g., [30]). Therefore, addressing stigma and enhancing mental health literacy can improve adolescents’ willingness to seek help and foster a supportive environment among peers, families, and communities [55].

This study aims to identify subgroups within the adolescent population based on their levels of knowledge about mental health, social stigma, experiences of intergroup anxiety, and endorsement of stereotypes related to mental health. We hope the findings can contribute to understanding the patterns and specific needs within this age group to inform targeted interventions that promote mental health education and reduce stigma surrounding mental health at young ages.

## 2. Materials and Methods

### 2.1. Participants

Participation in this study required the following inclusion criteria: being between 10 and 17 years old, willing to participate, possessing formal authorisation from their legal guardian, and having proficiency in reading Portuguese. On the other hand, the exclusion criteria included significant cognitive or learning impairments that might hinder the understanding of the survey.

The final sample consisted of 182 adolescents aged 10 to 17 (M = 13.8, SD = 2.4), of whom 50.6% were male. Most of the participants was in the 3rd cycle (41.2%), had Portuguese nationality (92.3%), and lived in the Central region of Portugal (38.5%). Regarding perceived social status, on a scale of 1 (lowest) to 10 (highest), an average of 6.6 (SD = 1.6) was reported. Regarding mental health problems, most participants said that they did not have mental health problems (87.1%) nor interact with people with mental health problems (86.3%).

### 2.2. Procedures

The present study is included in a broader project about knowledge and attitudes towards mental health, approved by the Ethics and Deontology Committee of the Psychology and Life Sciences of Lusófona University.

It employed a cross-sectional design with a quantitative methodology. Participants were recruited using non-probabilistic sampling methods, specifically convenience and snowball sampling, facilitated by flyers and social media outreach. The data was collected via an online survey conducted by Qualtrics.

The survey consisted of two sections. In the first section, the legal guardians of the adolescents were directed to a landing page featuring an informed consent form that provided a concise description of the project and its objectives. It also assured participants of data confidentiality, emphasizing that all responses were non-identifiable, and participation was voluntary, with the option to withdraw at any time. For the survey to proceed, legal guardians needed to consent to the participation of the adolescent under their care. In the second section, the adolescents were provided with the same information about the project and their involvement in the study, subsequently being asked to give their consent to participate.

### 2.3. Measures

The section of the survey addressed to legal guardians included questions about sociodemographic characteristics of the adolescent under their care, namely age, gender, nationality, region of residence, level of education, perceived social status, and diagnosis of mental health problems. In the section of the survey addressed to adolescents, the following instruments were used:

(1) Mental Health Knowledge: To measure Mental Health Knowledge, the Portuguese Version of Mental Health Knowledge Schedule was used [56,57]. This scale aims to assess and monitor knowledge about mental health, related to stigma. It is divided into two parts: six items that assess knowledge about mental health (e.g., “People with severe mental health problems can fully recover”) and six items that assess the ability to identify mental health problems (e.g., “Depression”). Participants answered on a 5-point Likert scale (1—strongly disagree—to 5—strongly agree), with higher scores indicating greater knowledge of mental health. In the validation for the Portuguese population, a Cronbach’s alpha of 0.29 was obtained for the total scale, 0.34 for the first part, and 0.20 for the second part [56]. In the present study, Cronbach’s alpha of 0.70 was obtained.
(2) Social Stigma: To measure social stigma, the Portuguese Version of AQ-8-C for children and adolescents [58,59] was used. This scale aims to measure social stigma towards people with mental health problems (e.g., “How scared of José would you feel”), rated on a 9-point Likert scale (1 = No or nothing to 9 = Very much or completely). Item 7 is reverse-coded, and the total score is obtained by averaging the items, with a higher score indicating greater social stigma. In the present study, Cronbach’s alpha of 0.70 was obtained.
(3) Intergroup Anxiety: The Intergroup Anxiety Scale [60], translated into the Portuguese language within the scope of this study, was used to evaluate emotions towards people with mental health problems. To translate the instrument, two translators fluent in Portuguese and English independently translated it into Portuguese. Then, both translations were compared, reaching an agreement on any discrepancies found to produce a preliminary version. Subsequently, two additional translators conducted a back-translation of the instrument into English, comparing it with the original version to identify any inconsistencies. Based on feedback from the project team, the final Portuguese version of the instrument was developed. Finally, this version was administered to the adolescent sample within the scope of the present study. It consists of seven items (e.g., “Anxious”), rated on a 7-point Likert scale (1 = I wouldn’t feel like that at all to 7 = I would feel like that). Items 3, 4, and 7 are reversed-coded. The scale score is obtained by summing the items, with a higher score indicating greater intergroup anxiety. In the present study, Cronbach’s alpha of 0.77 was obtained.
(4) Stereotypes about people with mental health problems: To measure negative stereotypes towards people with mental health issues [61,62], participants were presented with a list of 16 traits, reflecting warmth (e.g., “Dangerous”, “Friendly”) and competence dimension (e.g., “Capable”, “Competent”). Participants answered each trait on a 7-point Likert scale. Positive traits were reversed coded, and higher scores indicate greater endorsement of negative stereotypes. In the present study, Cronbach’s alpha of 0.89 was obtained.

### 2.4. Data Analysis

Data analysis was conducted using the IBM Statistical Package for Social Sciences (SPSS, version 30) to run descriptive and frequency analyses of sociodemographic data to characterise the sample. Cluster analysis was run in R statistical software, version 4.2.1 [63], using the stats package, to explore patterns of knowledge about mental health, social stigma, experiences of intergroup anxiety, and endorsement of negative stereotypes related to people who face mental health challenges.

Before clustering, the variables were standardised (z-scores) to ensure that variables with different scales contributed equally to the clustering solution. A two-step clustering procedure was performed. First, an agglomerative hierarchical cluster analysis was performed using the complete linkage method to explore initial clustering structures, using a Dendogram to establish an adequate number of clusters. The most favourable number of clusters was further confirmed using three more complementary methods: the Gap Statistic (Cluster package, [64]), the Elbow Method (factoextra package, [65]), and the Silhouette Method (Cluster package, [64]). This dual approach ensured both a theoretically informed and data-driven identification of clusters. Based on this, a K-means clustering algorithm was conducted to assign participants to the clusters, iteratively minimising within-cluster variance. This approach ensured a robust data-driven grouping of participants, capturing distinct patterns in their knowledge, attitudes, and beliefs related to mental health. The resulting cluster memberships were then used for further analysis and interpretation.

To complement the cluster analysis and provide a clear visualisation of the grouping structure, a Principal Component Analysis (PCA) was conducted using the factoextra package in R [65]. The PCA reduced the dimensionality of the data while retaining most of the variance. The first two principal components (PC1 and PC2) explained 65.4% of the variance (PC1: 37.8%, PC2: 27.6%), providing a meaningful framework for visualising the clusters in a two-dimensional space.

Following this visual exploration, to identify the characteristics of the clusters and to characterise the profile of the adolescents with each cluster, analysis of variance (ANOVA) and chi-square analysis were performed (both using the stats package [63]). Sensitivity power analysis for the ANOVA was conducted in G*Power 3.1 [66] with the following parameters: three groups, power of 0.95, alpha of 0.05, and a sample size of 182, establishing an effect size of 0.29. Additionally, the post hoc Tukey HSD test was conducted for the follow-up comparisons.

Firstly, an analysis of variance (ANOVA) was performed on the main outcomes to identify the characteristics of the clusters. Secondly, ANOVA analyses were also performed to compare the clusters on quantitative variables such as age and perceived social status. Chi-squared tests were used to compare categorical variables like gender (female vs. male), previous mental health status (Yes vs. No), and previous contact with people with mental health issues (Yes vs. No).

## 3. Results

We applied cluster analysis to identify patterns within our adolescent sample based on similarities in mental health knowledge, social stigma, intergroup anxiety, and endorsement of negative stereotypes.

Based on the dendrogram, a three-cluster solution was selected. The decision is supported by the substantial increase in height (distance) observed at the level of three clusters, indicating distinct separations between groups. Furthermore, the selection of (*k* = 3) clusters was supported by multiple methods. The Silhouette Method maximised the average silhouette width at 0.27, indicating good overall cluster separation. The Gap Statistic further validated this solution, which showed a peak at (*k* = 3), and the Elbow Method, revealed a clear inflection point at the same cluster number. These results confirm that a three-cluster solution effectively captures the structure in the data.

To further validate and explore the structure of the three-cluster solution, a Principal Component Analysis (PCA) was conducted to project the clusters onto a two-dimensional space. PC1 (variance explained 37.8%), labeled the Attitudinal Dimension, captured differences in intergroup anxiety and attributions, with strong contributions from Intergroup Anxiety (−0.64) and Social Stigma (−0.64). PC2 (variance explained 27.6%), termed the Knowledge Dimension, was dominated by mental health knowledge (−0.84), reflecting variations in understanding of mental health issues.

**Figure 1:**
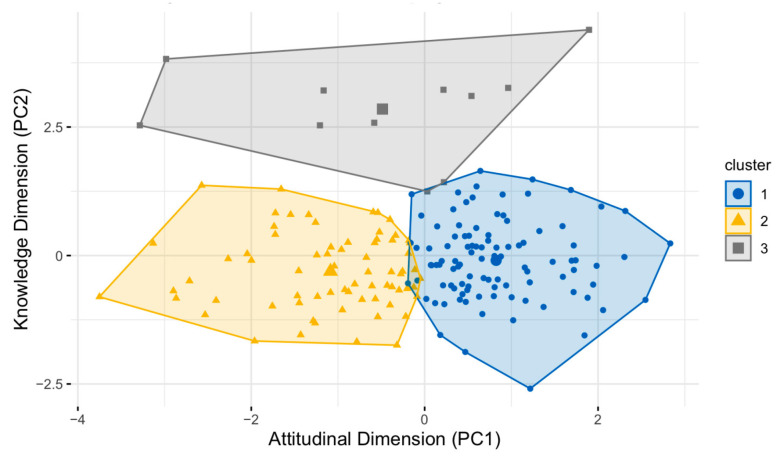
K-means clustering visualisation.

The PCA scatterplot (Figure 1) visually represents the clusters in the space defined by these two components. The clusters were separated: Cluster 1 showed more positive attitudes (higher values on PC1), while Cluster 3 demonstrated lower mental health knowledge (higher values on PC2). Cluster 2 occupied an intermediate position along PC1, reflecting ambivalent attitudes, and was distributed across PC2, indicating varying levels of mental health knowledge.

Following this visual exploration, the characteristics of each cluster were further analyzed. Table 1 describes the characteristics of each cluster in terms of the variables under study.

ANOVA results revealed significant differences across clusters in all variables, including intergroup anxiety (*F* (2, 179) = 101.10, *p* < 0.001, *η*^2^ = 0.53), mental health knowledge (*F* (2, 179) = 107.70, *p* < 0.001, *η*^2^ = 0.55), social stigma (*F* (2, 179) = 23.67, *p* < 0.001, *η*^2^ = 0.21), and stereotype endorsement (*F* (2, 179) = 21.47, *p* < 0.001, *η*^2^ = 0.19).

The first subgroup, Potential Advocates (55%; *n* = 100), was characterized by high levels of mental health knowledge, low social stigma, low intergroup anxiety, and moderate endorsement of stereotypes. The second subgroup, Ambivalents (39%; *n* = 71), exhibited high levels of mental health knowledge, moderate social stigma, elevated intergroup anxiety, and low endorsement of stereotypes. Finally, the third subgroup, Stigmatizers (6%; *n* = 11), demonstrated low levels of mental health knowledge, pronounced social stigma, moderate intergroup anxiety, and a high tendency to endorse stereotypes. The Tukey post hoc tests revealed several significant differences between the clusters. For Intergroup Anxiety, Potential Advocates reported significantly lower levels than both Ambivalents (*M*_diff_ = −13.74, *p* < 0.001) and Stigmatizers (*M*_diff_ = −8.20, *p* < 0.001), while Ambivalents also had significantly lower anxiety than Stigmatizers (*M*_diff_ = −5.54, *p* = 0.019). Regarding Mental Health Knowledge, Stigmatizers scored significantly lower than both Ambivalents (*M*_diff_ = −25.62, *p* < 0.001) and Potential Advocates (*M*_diff_ = −25.40, *p* < 0.001), with no significant difference between the latter two (*M*_diff_ = −0.21, *p* = 0.967). For Stereotypes Endorsement, Ambivalents reported significantly lower levels than both Potential Advocates (*M*_diff_ = −0.94, *p* < 0.001) and Stigmatizers (*M*_diff_ = 1.15, *p* = 0.001). No significant difference was observed between Potential Advocates and Stigmatizers (*M*_diff_ = 0.21, *p* = 0.770). Finally, for Social Stigma, Potential Advocates reported significantly lower levels than both Ambivalents (*M*_diff_ = −1.11, *p* < 0.001) and Stigmatizers (*M*_diff_ = 1.28, *p* = 0.001), with no significant difference between Ambivalents and Stigmatizers (*M*_diff_ = 0.18, *p* = 0.874).

Regarding the profiles of adolescents within each cluster in terms of their socio-demographic variables, a chi-squared test revealed a significant association between gender and cluster membership (χ^2^ (2) = 8.56, *p* = 0.014). Examining the frequencies, Ambivalents (57% females, *n* = 39, and 43% males, *n* = 30) and Potential Advocates (49% females, *n* = 47, and 51% males, *n* = 49) had a relatively balanced gender distribution. In contrast, Stigmatizers were predominantly male, with only 1 female and 10 males in the cluster. These results suggest that gender composition varied significantly across clusters, particularly for the Stigmatizers, where males were overrepresented. ANOVA results indicated no significant differences between clusters on age (*F* (2, 179) = 0.75, *p* = 0.473) and on perceived social status (*F* (2, 179) = 1.25, *p* = 0.289). When comparing the constitution of the clusters regarding previous experiences with mental health, results from chi-squared tests revealed no significant association between having mental health problems and cluster membership (χ^2^ (2) = 0.39, *p* = 0.823), suggesting that the prevalence of mental health problems was consistent across the clusters. Similarly, no significant association between living with someone with mental health problems and cluster membership (χ^2^ (2) = 2.40, *p* = 0.302), showing that the distribution of individuals living with someone with mental health problems was similar across all clusters.

## 4. Discussion

The current study had the objective of identifying adolescents’ subgroups based on their mental health knowledge, attitudes toward social stigma, experiences of intergroup anxiety, and endorsement of mental-health-related stereotypes. The findings provided insight into the spectrum of attitudes towards mental health, ranging from more supportive to more stigmatising. They identified three subgroups: (1) “Potential Advocates”, characterised by high levels of mental health knowledge, reduced levels of social stigma, low levels of intergroup anxiety, and moderate endorsement of stereotypes; (2) “Ambivalents”, marked by high mental health knowledge, moderate levels of social stigma, increased levels of intergroup anxiety, and low endorsement of stereotypes; and (3) “Stigmatizers”, defined by limited mental health knowledge, significant levels of social stigma, moderate levels of intergroup anxiety, and a tendency to endorse mental health-related stereotypes.

Overall, the results suggest that groups with higher levels of health knowledge report decreased social stigma, intergroup anxiety, and stereotypes. In comparison, the groups with lower levels of health knowledge tend to show increased social stigma, intergroup anxiety, and stereotypes. In line with these findings, scientific evidence shows that mental health literacy, the capacity to identify signs of mental health problems and to seek appropriate help, promote well-being, and support others facing challenges [34], is related to reduced stigma, intergroup anxiety, and stereotypes [67,68].

### 4.1. Potential Advocates

The first subgroup, Potential Advocates, revealed increased mental health knowledge, low social stigma, decreased intergroup anxiety, and moderate endorsement of mental-health-related stereotypes. Therefore, they may have been exposed to education or awareness programs that have equipped them with accurate information about mental health. According to the literature in this field, educating adolescents regarding mental health empowers them to challenge stigmatising beliefs, playing a pivotal role in reducing the stigma surrounding mental health [52,67]. Also, scientific evidence shows that low or poor mental health literacy is a major barrier to seeking professional help [69,70]. The competence or ability to recognise mental health problems is the first step towards seeking help [34]. In other words, high levels of mental knowledge indicate a good understanding of mental health disorders and their treatment options.

Low levels of social stigma suggest a more accepting and supportive attitude towards people with mental health problems, and the World Health Organisation [71] reinforces that in addition to personal attitudes, broad policies against discrimination are fundamental. There is evidence that public campaigns can reduce stigma by promoting literacy about mental health and that problems related to mental health are treatable. In addition, programs in schools and group interventions have shown positive results, promoting a supportive environment for seeking help and reducing fear of judgment [72].

Additionally, low levels of intergroup anxiety also suggest an accepting and supportive attitude towards people with mental health problems. Research indicates that intergroup anxiety can lead to avoidance behaviours, increased stigma, and negative stereotypes. Conversely, when individuals approach such interactions with confidence and comfort, the quality of these exchanges improves, contributing to reduced stigma and enhanced social integration for those with mental health problems. Positive contact experiences help dismantle biases and build empathy, fostering an atmosphere where individuals feel valued and understood [68,73].

Despite the subgroup showing limited levels of social stigma and intergroup anxiety, it still demonstrates moderate endorsement of mental-health-related stereotypes. A possible explanation for this result may be that while mental health knowledge contributes to a greater understanding of mental health issues, some stereotypes may persist due to deep-rooted social beliefs, lingering biases, or misconceptions about mental health [74].

### 4.2. Ambivalents

The second subgroup, Ambivalents, revealed increased mental health knowledge, moderate social stigma, profound intergroup anxiety, and decreased endorsement of stereotypes. Similar to the previous group, high levels of mental health knowledge suggest a great understanding of mental health, which may contribute to challenging biases and misconceptions and, consequently, reducing the endorsement of stereotypes [67].

Despite high mental health knowledge and low endorsement of stereotypes, this group still reveals signs of ambivalence, evidenced in heightened levels of intergroup anxiety and social stigma, suggesting discomfort or insecurity in social situations with people with mental health problems. These findings may indicate that some negative attitudes towards people with mental health problems persist, potentially driven by apprehension or personal fears that knowledge about mental health cannot eliminate [75].

Considering that some mental health problems are often associated with perceived risk or unpredictability, even in adolescents with high mental health literacy, this perception can foster intergroup anxiety and maintain a degree of social distance and stigmatisation towards people with mental health problems [37]. Such ambivalence can be further explained by cognitive dissonance, i.e., the conflict between mental health knowledge and entrenched societal stereotypes or personal biases, which may contribute to mixed attitudes and hinder full acceptance [67].

### 4.3. Stigmatizers

The third subgroup, Stigmatizers, revealed limited mental health knowledge, evident social stigma, moderate intergroup anxiety, and a tendency to stereotype endorsement. Contrary to the other two subgroups, the “Stigmatizers” evidenced low mental health literacy, suggesting an absence of understanding or awareness regarding mental health. In line with these findings, scientific evidence has shown that adolescents with limited knowledge of mental health problems are more prone to holding misconceptions and erroneous beliefs, which can be manifested in the endorsement of stereotypes, such as the perception of people with mental health problems as dangerous or unpredictable. Consequently, these stereotypes contribute to social stigma, leading to discrimination and social exclusion for affected individuals [52,74].

High levels of social stigma suggest negative attitudes towards people with mental health problems, which are related to misconceptions, lack of knowledge, or fear regarding mental health problems. The consequences of stigma surrounding mental health are profound, discouraging people from seeking help, reducing self-esteem, exacerbating isolation, and worsening health outcomes [23,49].

Low mental health literacy also results in greater intergroup anxiety, manifested in the apprehension or discomfort felt during interactions with people with mental health problems [60]. Intergroup anxiety may also lead to avoidance behaviours and less meaningful engagement, reinforcing negative attitudes and reducing opportunities for positive contact [34].

### 4.4. Contributions and Practical Implications

The present study performed a cluster analysis to identify patterns regarding mental health knowledge, social stigma, intergroup anxiety, and endorsement of stereotypes. The findings emphasize the diversity of adolescent perceptions concerning mental health and underscore the pivotal influence of knowledge, stigma, intergroup interactions, and stereotypes in shaping attitudes towards mental health.

Our findings can contribute to informing intervention and prevention efforts targeting mental health stigma in adolescents, which are crucial for fostering positive attitudes and ensuring long-term well-being. Adolescents are at a lifecycle stage of identity formation and have various perceptions, experiences, and attitudes based on cultural, social, and individual factors; interventions must be tailored to their specificities and everyday contexts. Interventions must be sensitive to their developmental stage and address topics, such as mental health, the impact of stigma, the importance of seeking help, peer influence, self-identity, and empathy, to reduce stigma, encourage help-seeking behaviours, and create more inclusive, supportive, and mentally healthier generations.

Promoting mental health literacy is a vital strategy for reducing social stigma, alleviating intergroup anxiety, and challenging stereotypes. It also has a critical role in fostering more inclusive and supportive communities [76]. The content of educational programs is a significant factor influencing the effectiveness of anti-stigma campaigns [77]. For instance, Salerno [78] found that most programs have successfully enhanced knowledge about mental health, reduced negative stereotypes, and increased motivation to seek professional help.

Structurally, stigma-reduction programs typically employ two main approaches: education—focused on deconstructing myths and providing accurate information about mental illness—and contact—featuring presentations by individuals with lived experiences of mental illness who share their personal stories [79].

Furthermore, integrating mental health literacy into school curricula helps normalise mental health as a routine aspect of daily life, reaching all students and creating a culture of openness. This approach also equips teachers with greater knowledge about youth mental health, enabling them to adequately support their students [80]. Several countries, such as Australia and the United Kingdom, have implemented national courses on mental health literacy [81]. In 2017, a program of study was developed for high schools, starting in 2022, in response to requests from academic institutions and mental health support groups. This course, aimed at young people aged between 15 and 18 years old, addresses the mechanisms of mental illness, typical symptoms, and self-help strategies, which encourages help-seeking and reduces mental health stigma [81].

Although high mental health literacy can help reduce erroneous beliefs and increase understanding about mental health, persistent societal norms, personal experiences, and deep-rooted biases can still lead to intergroup anxiety and social stigma, as shown in the higher mental health knowledge subgroups. Addressing these issues may require social contact interventions that facilitate positive interactions between adolescents and people with mental health problems can be beneficial in improving perceptions about people with mental health problems, creating more supportive environments [52], and reducing anxiety and negative perceptions by increasing familiarity and trust [68].

Also, addressing social stigma through peer-led initiatives and anti-stigma campaigns has been demonstrated to be effective in mitigating stigma associated with mental health and in promoting empathy, understanding, and acceptance among adolescents. Peer-led initiatives leverage relatable role models who can foster more open discussions and create a safe space for adolescents to engage in mental health [82]. Additionally, anti-stigma campaigns play an important role in raising awareness and changing attitudes towards mental health conditions [53]. These strategies, which often include educational content and personal testimonies, contribute to debunking myths and improving knowledge, attitudes, and behaviours around mental health [83]. On the other hand, the identification of groups through cluster analysis, namely stigmatised groups, allows psychologists to develop and implement specific interventions for each group, to reduce stigma and discrimination [84,85]. These interventions can include awareness campaigns, workshops, etc., that facilitate positive interactions and reduce prejudices [85].

### 4.5. Limitations and Future Studies

Despite the implications of the current study, limitations have also been identified. First of all, the cross-sectional design has advantages (e.g., simplicity, cost-effectiveness, shorter period for data collection, and a lower burden on participants), but also has disadvantages, such as the inability to separate a presumed cause from its possible effect—i.e., two concepts may be correlated, but this does not mean that one causes the other; it is necessary to demonstrate that the “cause” precedes its “effect” in time [86]. In this sense, it would be important to carry out a longitudinal study in the future to assess changes in attitudes, perceptions, and behaviours among adolescents.

The sample is not representative of the population; therefore, it is important to have a bigger and more diverse sample, from various demographic backgrounds, including different age groups, socioeconomic statuses, cultural backgrounds, and geographic regions in future studies.

A limitation of this study is the broad age range of the sample, encompassing early to late adolescence. However, no significant differences in age were observed across the clusters, suggesting that patterns of stigma, intergroup anxiety, and mental health knowledge are consistent throughout this developmental period. Nevertheless, future research with larger and diverse samples could further examine whether developmental differences play a more prominent role in shaping these outcomes and inform age-specific intervention strategies if needed.

The instruments used are self-report instruments, which may have some inaccuracy in the answers due to the participants’ misinterpretation of the questions [87] caused, not only by their perceptions, but also their characteristics, and may not fully capture the complexity of adolescents’ attitudes, which may bias the results of the study. According to Dang et al. [88], self-report measures ask participants to reflect on their behaviour in a variety of real-life situations, as well as asking them to reflect on how they usually behave. In future studies, it would be important to use mixed methods approaches to have deeper insights into adolescents’ experiences, perspectives, and lived realities. AQ-8-C (Abbreviated Attribution Questionnaire for Children) also provided ambiguous results, indicating that the questionnaire’s outcomes were unclear or inconclusive. This can be a significant limitation in understanding and addressing the attitudes being measured. Plus, the cluster “Stigmatizers” is small, making it difficult to detect significant patterns or relationships, which may limit the reliability of findings. It is therefore essential to consider that the sample size calculation is fundamental for the research to have adequate power to show clinically significant differences [89]. As such, it will be necessary to guarantee a larger sample in order to ensure adequate statistical power to improve not only the reliability but also the replicability of the study [90]. Additionally, deciding whether subgroups exist in the data can have important theoretical and clinical consequences (e.g., when group analysis is used as a data-driven approach to define diagnostic groups) or patient groups in clinical practice [91,92].

## Figures and Tables

**Table 1:** Cluster Profiles Based on Key Variables: Means and ANOVA Results.

	Potential Advocates (55%; *n* = 100)		Ambivalents (39%; *n* = 71)		Stigmatizers (6%; *n* = 11)		
	M	SD	M	SD	M	SD	*F*
Mental Health Knowledge	43.04 a	5.86	43.25 a	4.64	17.64 b	8.21	107.70 ***
Social Stigma	2.51 a	0.79	3.62 b	1.26	3.79 b	2.13	23.67 ***
Intergroup Anxiety Scale	17.53 a	5.93	31.27 c	5.65	25.73 b	11.28	101.10 ***
Stereotype Scale	4.77 a	1.01	3.83 b	0.83	4.98 a	1.42	21.67 ***

Note. Significant differences existed between groups on all variables (*** *p* < 0.001). Different letters within a row indicate significantly different means on post hoc HSD Tukey tests.

## Data Availability

Data will be made available upon reasonable request.
